# Introduction to the Special Issue on Advances in Pediatric and Adolescent Psychosocial Oncology

**DOI:** 10.3390/cancers17182957

**Published:** 2025-09-10

**Authors:** Lori Wiener, Amanda Thompson

**Affiliations:** 1 Pediatric Oncology Branch, Center for Cancer Research, National Cancer Institute, National Institutes of Health, Bethesda, MD 20892, USA; 2 Life with Cancer, Inova Schar Cancer Institute, Fairfax, VA 22031, USA; althomps412@gmail.com

Psychosocial care is critical to supporting the adjustment, coping, and quality of life of children and families from the time of diagnosis throughout treatment and into survivorship or through end of life and bereavement. Now a standard of care in pediatric oncology [1], psychosocial support is considered essential for all children and families. Interprofessional team members collaborate in assessment, interventions, education, and research to advance the science and practice of pediatric psycho-oncology.

The field of pediatric and adolescent psycho-oncology continues to evolve, with new research and innovative interventions. New insights are critical for improving the overall care and long-term outcomes for patients and their families and driving future research in pediatric psycho-oncology. Within this Special Issue, recent innovations in the field are described from a clinical and scientific point of view.

Three papers in this Special Issue present data on caregivers’ needs, an emerging and critical area of study that recognizes the impact of diagnosis and treatment on the entire family unit. In “*Somewhat of an Adult”: Understanding the “Dance” of Competing Tensions Parents Manage While Caring for an Adolescent or Young Adult (AYA) Diagnosed with Hematologic Malignancy*” (https://www.mdpi.com/2072-6694/17/8/1299), Mullis et al. [2] describe how they conducted in-depth interviews with 20 parents. Tensions were identified regarding the “dance” of being the parent and caregiver of an AYA during the developmental stage when they are seeking more independence. The authors recommend psychosocial education that normalizes these tensions and teaches parents to navigate them in ways that enhance connection with their AYA.

In “*Psychosocial Outcomes in Parents of Children with Acute Lymphoblastic Leukaemia in Australia and New Zealand Through and Beyond Treatment*” (https://www.mdpi.com/2072-6694/17/7/1238), Parker et al. [3] present the results of a prospective longitudinal study conducted across eight sites in Australia and New Zealand. The Emotion Thermometer (ET) tool and Patient-Reported Outcome Measurement Information System (PROMIS) questionnaires were used to quantify the psychological symptoms and need for help of parents whose children had been newly diagnosed with ALL. A total of 117 parents completed 327 surveys over a period spanning 0 to 62 months post-diagnosis. Their distress peaked within the first 6 months, with 40% of parents reporting clinically significant symptoms. Anxiety was the most consistently elevated symptom, with over 50% of responses being above the clinical cut-off and a second peak around the two-year mark (i.e., the time treatment ended). Depression and the parents’ need for help also peaked closer to the diagnosis and declined over time. In contrast, anger remained consistently present, with 27% reporting clinically significant scores across all the time points. While many other studies conducted outside Australia and New Zealand have found that a notable minority of parents continue to report elevated distress levels over time, this study identified a specific need for intervention throughout the ALL treatment trajectory.

A third study by Bemis et al. [4], “*Problem-Solving Skills Training for Parents of Children Undergoing Hematopoietic Stem Cell Transplantation*” (https://www.mdpi.com/2072-6694/17/6/930) used a mixed-methods approach to examine the feasibility and acceptability of a specific intervention to address distress in the caregivers of children receiving hematopoietic stem cell transplantation (HSCT). In an intensive HSCT setting, the study tested Bright IDEAS^®^, an evidence-based cognitive–behavioral problem-solving skills training intervention with demonstrated efficacy for caregivers of children newly diagnosed with cancer [5]. Caregivers were assigned Bright IDEAS^®^ with usual care or usual care alone. The intervention involved six-to-eight sessions which empowered caregivers to manage challenges throughout transplantation. The findings suggest that Bright IDEAS^®^ may be a promising way to help caregivers during their child’s HSCT.

This Special Issue also includes four papers addressing programmatic approaches to both program development and the provision of psychosocial support within a pediatric oncology setting. Three papers specifically address the implementation of the 15 published, evidence-based Standards for Psychosocial Care for Children with Cancer and their Families. A paper by Foster et al. [6], “*Development of an Evaluation Tool for Monitoring the Delivery of Psychosocial Care in Pediatric Oncology Settings*” (https://www.mdpi.com/2072-6694/17/9/1550), described how a pediatric oncology program utilized the standards as a foundation for psychosocial program development. Ongoing gaps in care were identified, along with the need for greater progress toward achieving these standards. Reviewing and analyzing the Pediatric Psychosocial Standard of Care Institutional Assessment Tool (Matrix) [7] further highlighted the need for program development, as well as the design of institutionally specific objective measures to monitor program improvements over time. The processes used by the authors set an example for other centers interested in evaluating how efficiently their programs currently implement psychosocial standards of care.

In the paper “*An Interprofessional Approach to Developing Family Psychosocial Support Programs in a Pediatric Oncology Healthcare Setting*” (https://www.mdpi.com/2072-6694/17/8/1342), Turner et al. [8] examine the framework provided by the Standards for the Psychosocial Care for Children with Cancer and the Pediatric Psychosocial Preventative Health Model (PPPHM). Employing both the standards and the PPPHM, the authors developed a comprehensive tiered approach, with input from both parent advisors and staff, to support the psychosocial needs of families in a pediatric oncology setting. This then led to the development of over ten new programs to enhance support for families facing pediatric cancer at the Universal, Targeted, and Clinical tiers. The paper illustrates how an interdisciplinary approach that combines the expertise and strengths of diverse disciplines with the perspectives of patients and families is critical to successfully providing support throughout the treatment trajectory.

Bernstein et al. [9] also looked at ways to align psychological care with the established evidence-based Standards of Care in their paper, “*Integrated Psychological Services in Pediatric Oncology: Caregiver Perspectives at Diagnosis*” (https://www.mdpi.com/2072-6694/16/18/3137). They evaluated the New Oncology Program in Psychology (NOPP), designed to provide psychoeducation about and anticipatory guidance for coping with diagnosis and treatment. Caregivers who participated in the NOPP felt more prepared and equipped with strategies to manage difficult emotions over time than those who did not, and those who completed a cognitive assessment also felt more informed and prepared to deal with the potential effects of diagnosis and treatment on the patient’s cognitive/academic functioning. The results highlight important domains for universal assessment and interventions at the time of a new cancer diagnosis and suggest that psychological services are associated with positive caregiver perceptions of feeling informed, prepared, and equipped for managing the psychosocial and cognitive impacts of disease and treatment.

Programs that combine conventional treatment with complementary therapies remain limited. In a retrospective study, “*Patient Acceptability of the First Integrative Pediatric Oncology Unit in Spain—The Pediatric Cancer Center Barcelona Experience*” (https://www.mdpi.com/2072-6694/17/2/222), Martínez García et al. [10] describe the feasibility of implementing such a program, the acceptance of the interventions, and early data on the various care activities. Acupuncture, aromatherapy, and reflexology had high acceptance rates and support the feasibility of implementing an Integrative Pediatric Oncology Unit within a patient-centered care model in a comprehensive pediatric cancer center in Spain.

The remaining papers within this Special Issue address different aspects of care. The first addresses germline genetic testing, something increasingly being integrated into pediatric oncology, with little known about its impact on the family unit. A paper by Van Hoyweghen et al., “*Family-Level Impact of Germline Genetic Testing in Childhood Cancer: A Multi Family Member Interview Analysis”* (https://www.mdpi.com/2072-6694/17/3/517) [11], describes the experience of six families who opted for germline genetic testing for a cancer predisposition. Germline genetic testing was generally viewed as a valuable and straightforward step in their child’s oncology trajectory, though parents found it difficult to distinguish its impact from that of the overwhelming stressors of their child’s cancer diagnosis and treatment. Several themes emerged from the interviews, included familial communication about genetic testing, differences in parental coping, feelings of guilt and forgiveness, and worries about the future health of the family. The authors suggest that proactively addressing these challenges could improve the support provided for and experience of families undergoing germline genetic testing for cancer predisposition.

A second paper addressed a unique aspect of family communication that has received limited attention—the characteristics of therapeutic parent–child communication. Son and Kim [12] examined 10 papers in their scoping review, “*Therapeutic Parent–Child Communication and Health Outcomes in the Childhood Cancer Context*” (https://www.mdpi.com/2072-6694/16/11/2152), and identified characteristics of therapeutic verbal and nonverbal communication. Positive psychological health outcomes included less distress, a lower level of PTSS, less internalization and externalization of symptoms, improved social–emotional competencies, better peer relationships, and more cooperation during the procedure at the individual level, while the family-level outcomes included increased family cohesion and adaptation. The authors noted the need for longitudinal studies to identify specific aspects of communication that predict better psychological outcomes.

It is well-known that adolescents and young adults with cancer have unique psychosocial needs that often go unmet by healthcare systems. Korenblum et al.’s study, “*Factors Affecting Psychosocial Distress in Adolescents and Young Adults with Cancer: BRIGHTLIGHT Cross-Sectional and Longitudinal Cohort Study Results*” [13], contributes to a deeper understanding of the risk of distress and protective factors in AYAs. The study examined a large cohort of patients (over the 3 years following their diagnosis) and provided a longitudinal description of their psychological outcomes, with depression improving over time but anxiety remaining stable. A unique aspect of this study was that a patient and public involvement group (Young Advisory Panel, YAP) helped to contextualize and interpret the study results during a series of online focus group discussions. Like many of the papers in this Special Issue, this study demonstrates the vital role of the participation and engagement of the patients themselves in research, as the importance of considering the patient experience cannot be overemphasized.

Fair et al. considered how to prepare youth with osteosarcoma for amputation, a topic not yet discussed in the literature. Their paper, “*“There are two healing processes in cancer care. There’s a physical healing and a mental adaptation process”: A Pilot Study for Preparing Children and Adolescents with Osteosarcoma for Limb Amputation*” [14], is based on in-depth structured interviews with nine survivors. The participants described the type of support and guidance they received before surgery, including contact with amputation-related organizations and exposure to tangible tools, such as a physical model of a knee joint. Their need for emotional support was found to be unmet, and when it was available, support from fellow amputees and surgeons was the most meaningful. This is the first paper to describe ways to provide holistic, patient-centered care throughout the amputation process.

Finally, two commentaries provide new insights into the field of pediatric and adolescent psycho-oncology. In the first, “*Leveraging the Patient and Family Voice in the Development of Patient Education: Supporting the Pediatric Oncology Experience*” (https://www.mdpi.com/2072-6694/17/7/1201)*,* Jones et al. [15] explore how patients and their families can be actively involved in the creation, assessment, and implementation of patient education materials, fostering a collaborative partnership between families and clinicians. Detailed and concrete examples of this collaboration are provided, including the creation of patient education materials and a podcast. The commentary emphasizes the importance of partnering with patients and families to ensure that their voices remain central to developing and implementing interventions.

In the second commentary, “*Bridging the Gap: Embedding Psychosocial Oncology Research into Comprehensive Cancer Care for Children and Young People*” [16], Sansom-Daly et al. describe a rare opportunity to develop comprehensive psychosocial programming. Located in Australia, Minderoo Children’s Comprehensive Cancer Centre will be the first of its kind in the southern hemisphere, providing state-of-the-science medical and psychosocial care for children and their families regardless of their background or location. Recognizing the need for integrated care and the barriers to providing it, similarly to Jones et al., the authors highlight the importance of investing in partnerships, including families in research, and reforming funding so that mental health support becomes a routine part of cancer treatment.

Collectively, the papers in this Special Issue shed light on a broad range of timely and clinically relevant topics within pediatric and adolescent psychosocial oncology. Several themes emerge across this body of work, including the centrality of caregiver support, the importance of aligning psychosocial services with established evidence-based standards, and the value of integrating diverse voices (particularly those of patients and their caregivers and siblings) into the development and delivery of psychosocial resources and interventions. These studies also underscore the unique psychosocial needs of adolescents and young adults, the evolving nature of communication and education in cancer care, and the feasibility of employing integrative care models that include complementary therapies. Clinically, this Special Issue calls for continued efforts to embed psychosocial care into the standard pediatric oncology workflow, ensuring timely access to support across the continuum of care and for all family members.

Future research should prioritize the use of longitudinal and prospective designs to better elucidate the psychosocial trajectories of patients and their families, especially as the current treatments evolve and new ones emerge. Greater inclusion of underrepresented populations and attention to vulnerable patient groups will be critical to improving equity and relevance in the field. Finally, integrating the voices of patients and their caregivers and siblings into research and clinical innovation must remain a guiding principle, ensuring that psychosocial care is responsive and rooted in lived experience. The articles in this Special Issue lay a strong foundation for this next phase of discovery, programmatic innovation, and patient- and family-centered care in pediatric and adolescent psycho-oncology.

## References

[1] Wiener L., Kazak A.E., Noll R.B., Patenaude A.F., Kupst M.J. (2015). Standards for the Psychosocial Care of Children with Cancer and Their Families: An Introduction to the Special Issue. Pediatr. Blood Cancer.

[2] Mullis M.D., Bylund C.L., Bagautdinova D., Bryan E.G., Sae-Hau M., Weiss E.S., Lagmay J.P., Fisher C.L. (2025). “Somewhat of an Adult”: Understanding the “Dance” of Competing Tensions Parents Manage While Caring for an Adolescent or Young Adult (AYA) Diagnosed with Hematologic Malignancy. Cancers.

[3] Parker C., Schilstra C.E., McCleary K., Martin M., Trahair T.N., Kotecha R.S., Ramachandran S., Cockcroft R., Conyers R., Cross S. (2025). Psychosocial Outcomes in Parents of Children with Acute Lymphoblastic Leukaemia in Australia and New Zealand Through and Beyond Treatment. Cancers.

[4] Bemis H., Ritter M., Lee M.N., Murray P., Noll R., Barber R., Balian C., Ward J. (2025). Problem-Solving Skills Training for Parents of Children Undergoing Hematopoietic Stem Cell Transplantation: A Mixed Methods Feasibility Study. Cancers.

[5] Sahler O.J., Dolgin M.J., Phipps S., Fairclough D.L., Askins M.A., Katz E.R., Noll R.B., Butler R.W. (2013). Specificity of problem-solving skills training in mothers of children newly diagnosed with cancer: Results of a multisite randomized clinical trial. J. Clin. Oncol..

[6] Foster K., Sadler B., Conrad A.L., Grafft A. (2025). Development of an Evaluation Tool for Monitoring the Delivery of Psychosocial Care in Pediatric Oncology Settings. Cancers.

[7] Wiener L., Kupst M.J., Pelletier W., Kazak A.E., Thompson A.L. (2020). Tools to guide the identification and implementation of care consistent with the psychosocial Standards of care. Pediatr. Blood Cancer.

[8] Turner E., Sirrine E.H., Crabtree V.M., Elliott D.A., Carr A., Elsener P., Parris K.R. (2025). An Interprofessional Approach to Developing Family Psychosocial Support Programs in a Pediatric Oncology Healthcare Setting. Cancers.

[9] Bernstein E., Jones A.M., Jurbergs N., Harman J.L., Phipps S., Heidelberg R.E. (2024). Integrated Psychological Services in Pediatric Oncology: Caregiver Perspectives at Diagnosis. Cancers.

[10] Martinez Garcia E., Lopez de San Roman Fernandez C., Nishishinya Aquino M.B., Perez-Jaume S., Fernandez-Jane C., Cruz Martinez O., Morales La Madrid A. (2025). Patient Acceptability of the First Integrative Pediatric Oncology Unit in Spain-The Pediatric Cancer Center Barcelona Experience: A Retrospective Study. Cancers.

[11] Van Hoyweghen S., Claes K.B.M., de Putter R., Wakefield C.E., Van Poucke M., Van Schoors M., Hellemans S., Verhofstadt L. (2025). Family-Level Impact of Germline Genetic Testing in Childhood Cancer: A Multi Family Member Interview Analysis. Cancers.

[12] Son H., Kim N. (2024). Therapeutic Parent-Child Communication and Health Outcomes in the Childhood Cancer Context: A Scoping Review. Cancers.

[13] Korenblum C., Taylor R.M., Fern L.A., Hough R., Wickramasinghe B. (2025). Factors Affecting Psychosocial Distress in Adolescents and Young Adults with Cancer: BRIGHTLIGHT Cross-Sectional and Longitudinal Cohort Study Results. Cancers.

[14] Fair C., Wurst B., Wiener L. (2025). “There Are Two Healing Processes in Cancer Care—There is a Physical Healing and a Mental Adaptation Process”: A Pilot Study for Preparing Children and Adolescents with Osteosarcoma for Limb Amputation. Cancers.

[15] Jones A.M., Marchetta A., Parris K.R., Heidelberg R.E., Jurbergs N. (2025). Leveraging the Patient and Family Voice in the Development of Patient Education: Supporting the Pediatric Oncology Experience. Cancers.

[16] Sansom-Daly U.M., McLoone J.K., Fardell J.E., Evans H.E., McGill B.C., Robertson E.G., Signorelli C., Ellis S., Ha L., Hetherington K. (2025). Bridging the Gap: Embedding Psychosocial Oncology Research into Comprehensive Cancer Care for Children and Young People. Cancers.

